# Comparison of molecular surveillance methods to assess changes in the population genetics of *Plasmodium falciparum* in high transmission

**DOI:** 10.3389/fpara.2023.1067966

**Published:** 2023-04-03

**Authors:** Anita Ghansah, Kathryn E. Tiedje, Dionne C. Argyropoulos, Christiana O. Onwona, Samantha L. Deed, Frédéric Labbé, Abraham R. Oduro, Kwadwo A. Koram, Mercedes Pascual, Karen P. Day

**Affiliations:** ^1^ Department of Parasitology, Noguchi Memorial Institute for Medical Research, University of Ghana, Legon, Ghana; ^2^ Department of Microbiology and Immunology, The University of Melbourne, Bio21 Institute and Peter Doherty Institute, Melbourne, VIC, Australia; ^3^ Department Ecology and Evolution, The University of Chicago, Chicago, IL, United States; ^4^ Navrongo Health Research Centre, Ghana Health Service, Navrongo, Ghana; ^5^ Epidemiology Department, Noguchi Memorial Institute for Medical Research, University of Ghana, Legon, Ghana; ^6^ Santa Fe Institute, Santa Fe, NM, United States

**Keywords:** population genetics, high transmission, SNPs (single nucleotide polymorphisms), microsatellies, *var* genes, molecular markers, malaria control interventions, *Plasmodium falciparum*

## Abstract

A major motivation for developing molecular methods for malaria surveillance is to measure the impact of control interventions on the population genetics of *Plasmodium falciparum* as a potential marker of progress towards elimination. Here we assess three established methods (i) single nucleotide polymorphism (SNP) barcoding (panel of 24-biallelic loci), (ii) microsatellite genotyping (panel of 12-multiallelic loci), and (iii) *var*coding (fingerprinting *var* gene diversity, akin to microhaplotyping) to identify changes in parasite population genetics in response to a short-term indoor residual spraying (IRS) intervention. Typical of high seasonal transmission in Africa, multiclonal infections were found in 82.3% (median 3; range 1-18) and 57.8% (median 2; range 1-12) of asymptomatic individuals pre- and post-IRS, respectively, in Bongo District, Ghana. Since directly phasing multilocus haplotypes for population genetic analysis is not possible for biallelic SNPs and microsatellites, we chose ~200 low-complexity infections biased to single and double clone infections for analysis. Each genotyping method presented a different pattern of change in diversity and population structure as a consequence of variability in usable data and the relative polymorphism of the molecular markers (i.e., SNPs < microsatellites < *var*). *Var*coding and microsatellite genotyping showed the overall failure of the IRS intervention to significantly change the population structure from pre-IRS characteristics (i.e., many diverse genomes of low genetic similarity). The 24-SNP barcode provided limited information for analysis, largely due to the biallelic nature of SNPs leading to a high proportion of double-allele calls and a view of more isolate relatedness compared to microsatellites and *var*coding. Relative performance, suitability, and cost-effectiveness of the methods relevant to sample size and local malaria elimination in high-transmission endemic areas are discussed.

## 1 Introduction

The World Health Organization’s malaria elimination strategy recommends the use of molecular methods for surveillance to measure the impact of malaria control interventions on the population genetics of *Plasmodium falciparum* (World Health Organization, 2022). High-transmission endemic areas present a specific challenge for molecular surveillance, especially in sub-Saharan Africa (SSA) where ~95% of the global malaria cases occurred in 2021 (World Health Organization, 2022). Here there is extensive genomic diversity of the parasite population (e.g., Pf6 database (MalariaGEN Consortium, 2021)) with many infected individuals carrying multiclonal *P. falciparum* infections (i.e., complexity or multiplicity of infection (MOI) > 1) (Anderson et al., 2000; Mobegi et al., 2012; Auburn and Barry, 2017) with a genetic structure consistent with frequent sexual recombination (meiosis) (Babiker et al., 1994; Paul et al., 1995). This contrasts with the genetic diversity seen in low-transmission regions of South America and Southeast Asia, as well as areas of intense malaria control in SSA such as in Senegal and Zambia, where parasite populations are largely clonal (i.e., MOI = 1) and highly related (Anderson et al., 2000; Anthony et al., 2005; Pumpaibool et al., 2009; Daniels et al., 2013; Daniels et al., 2015; Noviyanti et al., 2015; Auburn and Barry, 2017).

Panels of multiallelic microsatellites have been widely used to look at *P. falciparum* population genetics in a range of transmission settings to define linkage disequilibrium (Anderson et al., 2000; Machado et al., 2004; Anthony et al., 2005; Mobegi et al., 2012; Yalcindag et al., 2012; Barry et al., 2013; Vera-Arias et al., 2019; Kattenberg et al., 2020). Most recently, biallelic single nucleotide polymorphisms (SNPs) have been used by malariologists working in low- to moderate-transmission settings to look at diversity and population structure of clinical infections in response to interventions (Daniels et al., 2013; Nkhoma et al., 2013; Daniels et al., 2015; Bei et al., 2018). Data from both SNPs and microsatellites were analyzed by neutral theory (Anderson et al., 2000; Daniels et al., 2008). However, when used in high transmission, SNP barcoding and microsatellite genotyping have limitations for genetic diversity inferences as multiclonal infections are common, resulting in multilocus haplotypes that cannot be accurately reconstructed or phased from genotyping data. Two empirical solutions have been proposed to analyze SNP and/or microsatellite data for population genetics from these complex infections. The simplest solution is to only use monoclonal *P. falciparum* infections, thereby reducing the sample size. Consequently many samples are collected to analyze the few (Daniels et al., 2013; Daniels et al., 2015; Verity et al., 2020). The alternative is to use multiclonal infections to identify the major allele at each of the SNP or microsatellite loci to infer or construct a “dominant infection haplotype” dataset (Anderson et al., 2000; Verity et al., 2020). More recently, various computational methods have been developed to infer haplotypes (Zhu et al., 2018; Zhu et al., 2019). These remain largely untested on datasets from high transmission and do not account for the rate of sexual recombination seen in moderate- to high-transmission endemic areas (Babiker et al., 1994; Paul and Day, 1998).

To characterize *P. falciparum* population structure in response to interventions, we have developed an empirical approach known as *var* genotyping or *var*coding based on *var* genes (~40-60 *var* genes per genome) which encode for the major variant surface antigen of asexual blood stages known as *P. falciparum* erythrocyte membrane protein 1 (PfEMP1) (Bull et al., 1998; Gardner et al., 2002; Rask et al., 2010; Otto et al., 2019). *Var*coding is a fingerprinting method by amplicon sequencing, akin to microhaplotyping, which identifies the diversity of the *var* genes per *P. falciparum* infection (i.e., isolate) using sequences encoding the immunogenic Duffy-binding-like α domain (DBLα) of PfEMP1 variants, defined as DBLα types. Analysis of the relationship between DBLα types and exon 1 of *var* genes in Malawi and Ghana has shown that each DBLα type, especially upsB and upsC types (i.e., non-upsA), is predominantly associated with a single *var* gene, and therefore DBLα type diversity acts as a suitable surrogate for *var* diversity per host and in the population (Tan et al., 2023). Prior population investigations based on DBLα types in high-transmission settings of SSA have demonstrated that sequences of this marker are highly diverse in local endemic areas with thousands of variants described (Barry et al., 2007; Chen et al., 2011; Day et al., 2017; Ruybal-Pesántez et al., 2017b; Rorick et al., 2018; Tonkin-Hill et al., 2021; Ruybal-Pesántez et al., 2022). Parasite genomes in natural populations in SSA are typically composed of distinct sets of *var* genes (i.e., *var* repertoires), which are largely non-overlapping (Chen et al., 2011; Day et al., 2017; Ruybal-Pesántez et al., 2017b; Rorick et al., 2018; Ruybal-Pesántez et al., 2022) likely due to immune selection (He et al., 2018). This makes it possible to count the number of diverse *var* repertoires (termed MOI*_var_*) present in an isolate by simply counting the number of DBLα types in an isolate (repertoire size) and then dividing by the median number of DBLα types amplified per genome (Ruybal-Pesántez et al., 2022; Tiedje et al., 2022). The method has been shown to work well across MOI ranges from 1 to > 20 (Labbé et al., 2023). *Var*coding does not require the construction of multilocus haplotypes for each clone in an isolate (i.e., phasing) due to the non-overlapping population structure of *var* repertoires allowing multiple *P. falciparum* clones to accumulate within a human host in high transmission (Barry et al., 2007; Chen et al., 2011; Day et al., 2017; Ruybal-Pesántez et al., 2017b; Ruybal-Pesántez et al., 2022). Using this method, measures of heterozygosity and similarity of isolate repertoires are easily calculated by the pairwise type sharing (PTS) statistic, a measure of identity-by-state (IBS) (Barry et al., 2007; Speed and Balding, 2015).

For molecular surveillance to be used routinely in Africa, it should efficiently report changes in diversity and population structure of *P. falciparum* locally and be easily deployed in endemic areas, especially in high transmission, in a cost-effective way. This requires us to identify genotyping techniques that provide information with few genetic markers to analyze relatively small sample sizes in regional laboratories. Here we assess the performance of three field applicable genotyping methods, i.e., (i) SNP barcoding (panel of 24-biallelic loci), (ii) microsatellite genotyping (panel of 12-multiallelic loci), and (iii) *var*coding under the conditions of high seasonal transmission in Ghana, one of the 11 highest burden countries for malaria globally (World Health Organization & Roll Back Malaria Partnership to End Malaria, 2019; World Health Organization, 2021). The effectiveness of these methods to describe changes in the diversity and similarity of *P. falciparum* at the end of the wet season before (October 2012) and after (October 2015) the implementation of three rounds of indoor residual spraying (IRS) using non-pyrethroid insecticides, managed under operational conditions (Tiedje et al., 2022) is documented. The IRS reduced transmission intensity in Bongo by > 90% at the peak of the wet season as measured by the monthly entomological inoculation rate (EIR) (infective bites/person/month (ib/p/m)) between August 2013 (pre-IRS) (EIR = 5.3 ib/p/m) and August 2015 (post-IRS) (EIR = 0.4 ib/p/m) (Tiedje et al., 2022). Coincident with this decrease in transmission, declines in microscopic *P. falciparum* prevalence (42.0% to 27.0%) and median densities (520 parasites/μL to 320 parasites/μL) were also observed pre- to post-IRS (Tiedje et al., 2022).

Each genotyping method presented a different pattern of change of population diversity and structure when sampling monoclonal or low-complexity infections, consistent with marker variability. The 24-SNP barcode was least informative, largely due to the biallelic nature of SNPs leading to a high proportion of double-allele calls (DACs), whereas microsatellites showed high haplotype diversity with ten markers but no measurable change in population structure after the IRS. For this sample size of 200 isolates, *var*coding and microsatellite genotyping provided the most informative analysis showing the overall failure of the IRS intervention to significantly change the population structure from pre-IRS (i.e., many diverse genomes of low genetic similarity). Although we note that *var*coding did detect a subtle shift towards less similarity of *var* repertoires after IRS suggesting the method is sensitive to decreased transmission creating fewer recombinant genomes as a consequence of less outcrossing. The results of this study provide useful information for high-burden countries in Africa looking at strategies to deploy molecular surveillance to assess changes in parasite diversity and population structure in relatively small sample sizes from sentinel sites in local endemic areas.

## 2 Materials and methods

### 2.1 Study population and ethical approvals

The *P. falciparum* data utilized in this study was collected from participants with microscopically confirmed asymptomatic *P. falciparum* infections (i.e., isolates) at the end of the wet season (i.e., high-transmission season) from two proximal catchment areas (i.e., Vea/Gowrie and Soe, with a sampling area of ~60 km^2^) in Bongo District, Ghana (hereinafter referred to collectively as “Bongo”) (Tiedje et al., 2022). Using an interrupted time-series study design, two age-stratified surveys of ~2,000 participants per survey were undertaken pre-IRS (October 2012) and post-IRS (October 2015) against a backdrop of widely distributed long-lasting insecticidal nets (LLIN) (Tiedje et al., 2022). Bongo, located in the Upper East Region, is categorized as a high-transmission area based on the WHO “A Framework for Malaria Elimination” (WHO/GMP, 2017) where *P. falciparum* prevalence was ≥ 35% in 2012 (73.8%) and 2015 (41.6%) (Tiedje et al., 2022). Detailed information on the study site, the study population, inclusion/exclusion criteria, data collection procedures, etc. have been described previously (Tiedje et al., 2017; Tiedje et al., 2022). The study was reviewed/approved by the ethics committees at the Navrongo Health Research Centre (Navrongo, Ghana), Noguchi Memorial Institute for Medical Research (Legon, Ghana), The University of Chicago (Chicago, United States), and The University of Melbourne (Melbourne, Australia).

### 2.2 Genotyping methods

#### 2.2.1 *Var*coding

For *var*coding, the sequences encoding the DBLα tags of *P. falciparum var* genes were amplified by PCR, pooled, and sequenced on the Illumina MiSeq platform (2x300bp paired-end configuration) (New York University Genome Technology Center, New York, NY, United States; Australian Genome Research Facility, Melbourne, Australia) (Day et al., 2017; Ruybal-Pesántez et al., 2017b; He et al., 2018; Ruybal-Pesántez et al., 2022). This high-throughput sequencing method was utilized for all participants with microscopically confirmed asymptomatic *P. falciparum* infections in the pre-IRS (N = 808) and post-IRS (N = 545) surveys (**Figure S1**, **Table S1**). The DBLα sequence tags were then cleaned, clustered, and classified using a suite of custom bioinformatic pipelines (Ruybal-Pesántez et al., 2017b; He et al., 2018). For a detailed description of these pipelines please see the tutorial: https://github.com/UniMelb-Day-Lab/tutorialDBLalpha.

#### 2.2.2 24-SNP molecular barcoding

The 24-SNP molecular barcoding was undertaken for the isolates selected for this comparative analysis (**Table S2**) using a 384-well format using the method described by Daniels et al. (2008). Briefly for each reaction, template and water in a total volume of 2.5 μl was added to a 2.5 μl mix made up of 0.125 μl 40× SNP assay and 2.5 μl Master Mix in a 384-well optical PCR plate and mixed, for a total reaction volume of 5 μl. The plate was covered with an optical plate seal and amplified in an ABI 7900 HT (Department of Parasitology, Noguchi Memorial Institute for Medical Research, Legon, Ghana). Following the amplification, all isolates were analyzed using the Applied Biosystem’s proprietary Allelic Discrimination and Absolute Quantitation software.

#### 2.2.3 Microsatellite genotyping

Microsatellite genotyping and sequencing methods utilized for the isolates included in this analysis (**Table S2**) have been previously genotyped for ten putatively neutral microsatellite markers (2490, TA81, TA87, TA109, TA60, POLYA, ARA2, PfG377, PfPK2, and TA40) (Argyropoulos et al., 2021).

### 2.3 Multiplicity of infection

Multiplicity of infection by *var*coding (i.e., MOI_*var*_) was calculated based on the number of DBLα types in an isolate due to the limited overlap between DBLα isolate repertoires as previously shown, especially in high transmission (Day et al., 2017; Ruybal-Pesántez et al., 2017b; Ruybal-Pesántez et al., 2022; Tiedje et al., 2022). To calculate MOI_*var*_ the non-upsA DBLα types were chosen since not only are they more diverse and less conserved than the upsA DBLα types, but they have also been shown to have a more specific 1-to-1 relationship with a single *var* gene compared to upsA (Tan et al., 2023). Based on each isolate’s non-upsA DBLα repertoire size, MOI*_var_* was estimated using a cut-off value of 45 non-upsA DBLα types per *P. falciparum* genome. This cut-off was selected based on the median number of non-upsA DBLα types identified for the 3D7 laboratory isolate included as a control during *var*coding (**Figure S2**). Using this cut-off, MOI_*var*_ bins were defined as follows: 1 to 45 non-upsA DBLα types were estimated to be monoclonal infections (i.e., MOI*_var_* = 1), isolates with 46 to 90 non-upsA DBLα types were estimated to be carrying two *P. falciparum* clones (i.e., MOI*_var_* = 2, multiclonal infections), isolates with 91 to 135 non-upsA DBLα types were estimated to be carrying three *P. falciparum* clones (i.e., MOI*_var_* = 3, multiclonal infections), and so on.

**Figure 1:**
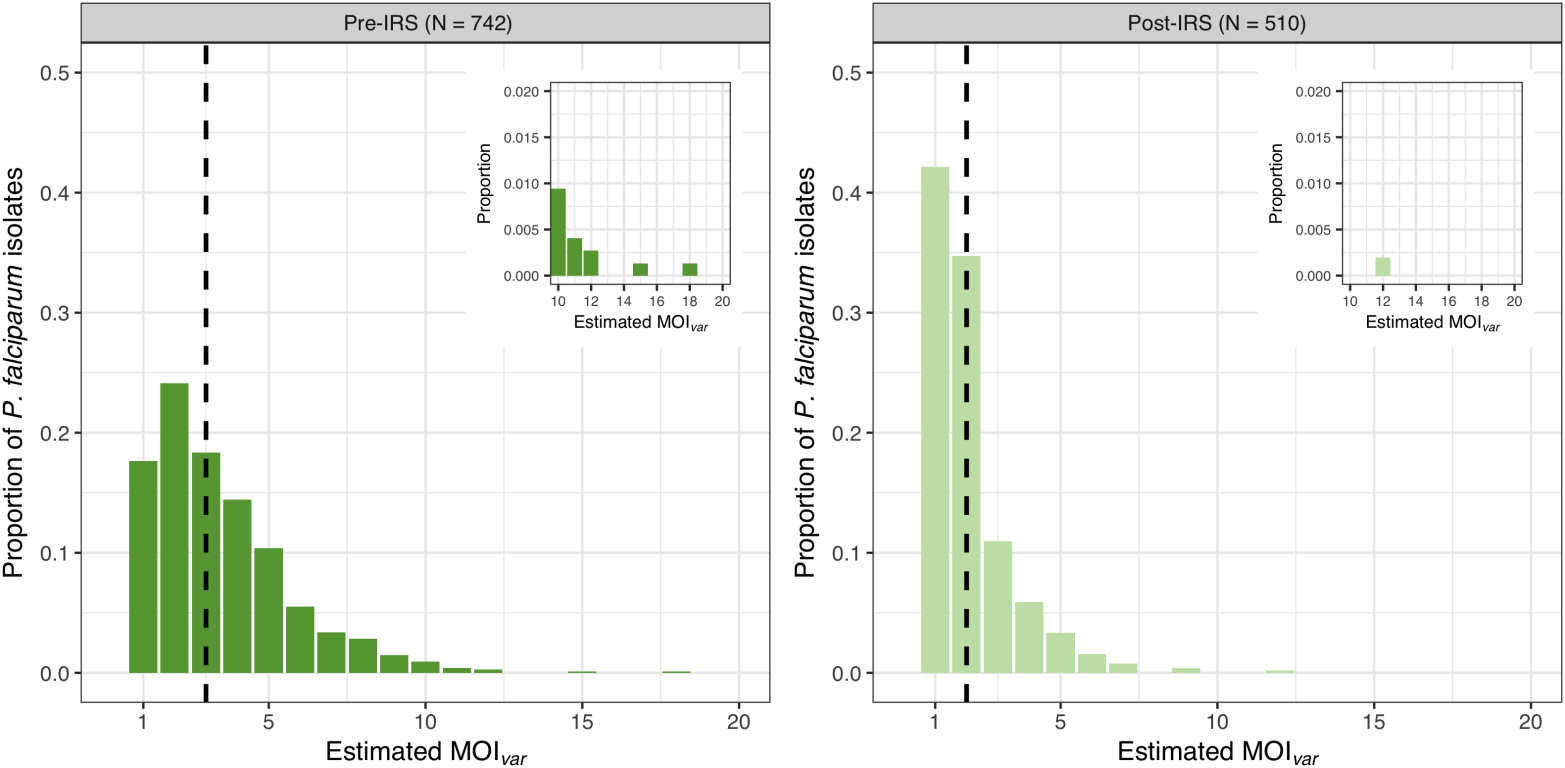
MOI*_var_* frequency distributions for all *P. falciparum* isolates with DBLα sequence data collected pre-IRS (dark green) and post-IRS (light green). The median MOI*_var_* pre-IRS (median = 3 [IQR: 2 – 5]) and post-IRS (median = 2 [IQR: 1 – 2]) are indicated with the black dashed lines. On the horizontal axis are the MOI*_var_* categories as determined using *var*coding (see Materials and Methods) for all *P. falciparum* isolates with DBLα sequence data (pre-IRS N = 742; post-IRS N = 510) (**Figure S1**; **Table S1**). The MOI*_var_* categories between 10 to 20 are shown in the upper right inserts to show the maximums for the pre-IRS (range: 1-18) and post-IRS (range: 1-12) surveys.

MOI*_var_* could only be estimated for those isolates with DBLα sequence data, resulting in 742 (91.8%) and 510 (93.6%) isolates in the pre- and post-IRS surveys, respectively (**Figure S1** and **Table S1**). For these isolates with DBLα sequencing data, the median asymptomatic *P. falciparum* densities in the pre-IRS (560 parasites/μL) and post-IRS (360 parasites/μL) surveys were of similar magnitudes and ~5-9 times higher compared to those isolates with no DBLα sequencing data pre-IRS (160 parasites/μL) and post-IRS (40 parasites/μL). Consistent with other high-transmission areas in SSA, we found that the majority of *P. falciparum* isolates were composed of multiclonal infections, i.e., 82.3% and 57.8% for the pre- and post-IRS surveys, respectively (**Figure 1**). Whilst the median MOI*_var_* values were 3 [IQR: 2 – 5] pre-IRS and 2 [IQR: 1 – 2] post-IRS, the MOI*_var_* frequency distributions show many individuals had multiclonal infections greater than three (39.9% and 12.1% pre- and post-IRS, respectively). At the extreme of the range, MOI*_var_* detected a maximum of 18 and 12 P*. falciparum* coinfections per isolate for the pre- and post-IRS, respectively (**Figure 1**).

### 2.4 Isolate filtering

In order to compare the performance of the three genotyping methods to describe diversity and population structure, a subset of 215 and 200 *P. falciparum* isolates with the lowest complexity were selected pre- and post-IRS, respectively. These isolates had previously been chosen for microsatellite genotyping using *var*coding to estimate MOI (Argyropoulos et al., 2021). They were then used for SNP genotyping. To improve the likelihood of obtaining SNP and microsatellite genotyping data, isolates with monoclonal infections (MOI*_var_* = 1) and low quality DBLα sequencing data (22% and 49% pre- and post-IRS, respectively) were not included in the subset of low-complexity isolates selected pre-IRS (N = 215, median MOI*_var_* = 2 [IQR: 1 – 2]) and post-IRS (N = 200, median MOI*_var_* = 1 [IQR: 1 – 2] (**Figure S1**; **Table S2**). Note that these isolates selected pre- and post-IRS were not statistically different than those isolates in the original study population for any of the key variables, except age pre-IRS (*p-value* < 0.001, Chi-square test) and parasitemia post-IRS (*p-value* < 0.01, Mann Whitney U test) (Tiedje et al., 2022).

### 2.5 Measures of genetic diversity

To estimate genetic diversity, the number of unique multilocus haplotypes (*h*), the number of alleles (*A*), and expected heterozygosity (*H_e_*) were calculated for the SNP and microsatellite markers using the R package *poppr* 2.9.3 (Kamvar et al., 2014; Kamvar et al., 2015). For the DBLα types, diversity was measured using richness, defined as the number of unique DBLα types observed (i.e., DBLα type pool size). In addition, we also assessed *var* (or DBLα type) expected heterozygosity (*H_v_*) (Rorick et al., 2018). If each isolate had a repertoire of exactly one DBLα type, pairwise type sharing (PTS) (see section 2.6 below for more information) would be roughly equivalent to *var* expected homozygosity (1 - *H_v_*), and thus by calculating the pairwise type difference (PTD = 1 - PTS) statistic we can obtain *var* expected heterozygosity (*H_v_*).

### 2.6 Measures of genetic similarity

Genetic similarity among the isolates in the pre- and post-IRS surveys, i.e., the number of shared loci (SNPs, microsatellites, and DBLα types), was assessed by comparing every isolate to every other isolate. To undertake the comparisons for the SNPs and microsatellites, we used the pairwise allele sharing (P_AS_) statistic, using only isolates with the “monoclonal infections” and complete multilocus infection haplotypes (i.e., no missing genotyping data) (Ruybal-Pesántez et al., 2017a; Argyropoulos et al., 2021). These complete multilocus haplotypes (i.e., phased isolates) for the SNPs and microsatellites were necessary to ensure that the denominator in the P_AS_ calculations would be consistent for the SNPs (i.e., 20 loci) and microsatellites (i.e., ten loci). The P_AS_ scores were calculated using *P_AS_ = n_ab_/n_l_*, where *n_ab_* is the number of alleles shared between the isolate haplotypes and *n_l_* is the number of loci examined (i.e., 20 SNPs and ten microsatellites). To measure similarity for the DBLα types we used the PTS statistic, calculated as *PTS = 2n_ab_/(n_a_ + n_b_)*, where *n_a_* and *n_b_* are the number of unique DBLα types in the repertoires of isolate *a* and isolate *b*, and *n_ab_* are the number of DBLα types shared between isolate *a* and isolate *b* (Barry et al., 2007). The advantages of P_AS_ and PTS is that they are convenient statistics that can be quickly and easily calculated to evaluate the number of SNP/microsatellite alleles or DBLα types shared between two different isolates. Both the P_AS_ and PTS scores were calculated between all isolate pairs in each survey (pre-IRS and post-IRS) and represent the proportion of sharing (or relatedness) between two isolates with scores ranging from 0 (i.e., dissimilar or unrelated) to 1 (i.e., identical or clones). Both the P_AS_ and PTS statistics are measures of identity-by-state (IBS) used to assess similarly or relatedness between isolates and were not used to infer inheritance from a recent common ancestor (i.e., identity-by-decent (IBD)) (Speed and Balding, 2015).

### 2.7 Statistical analysis

All statistical analyses were carried out in R 4.0.5 (Core Team, 2018) implemented in RStudio 1.4.1106 (RStudio, 2020) using the R package *tidyverse* 1.3.1 (Wickham et al., 2019) for data curation, analysis, and visualization.

## 3 Results

### 3.1 Genotyping of asymptomatic *Plasmodium falciparum* infections

Selection of isolates from asymptomatic individuals for genotyping was biased towards low-complexity infections as outlined in the Materials and Methods. The amount of usable data for each marker from these low-density asymptomatic infections was assessed for each marker as follows.

#### 3.1.1 SNPs

**Figure 2:**
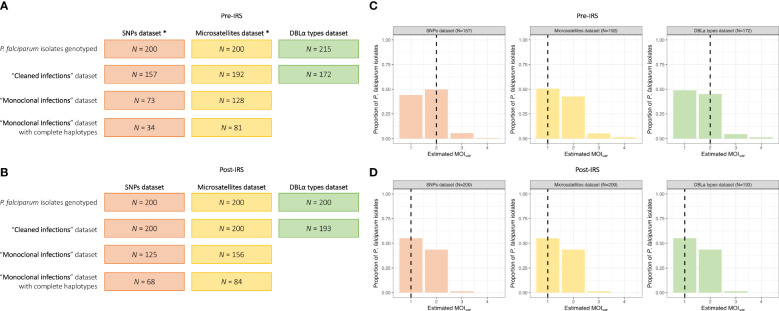
MOI*_var_* frequency distributions for the pre- and post-IRS surveys. Breakdown of the *P. falciparum* isolates selected and genotyped in the SNP (orange), microsatellite (yellow), and DBLα type (green) datasets used for the population genetic anlyses in the pre- **(A)** and post-IRS **(B)** surveys (**Table S2**). The “cleaned infections” datasets include only isolates with genotyping data meeting the established selection criteria for the SNPs (i.e., missing calls at ≤ 20% of the 20-SNP loci), microsatellites (i.e., data at ≥ 3 of the 10-microsatellite loci), and DBLα types (i.e., ≥ 20 DBLα types) (*Note: Of the 215 isolates selected pre-IRS, a slightly different subset of 200 isolates had to be used for the SNP and microsatellite genotyping due to isolate availability. However, between these two datasets, 92.5% (N = 185) isolates genotyped were the same.). To undertake the population genetic analyses, specifically for the SNPs and microsatellites, only isolates with “monoclonal infections” were used. Finally, to undertake the genetic similarity analyses using the SNPs and microsatellites, only those isolates with “monoclonal infections” and complete multilocus infection haplotypes (i.e., no missing genotype data, see Materials and Methods) were included. MOI*_var_* frequency distributions of the *P. falciparum* isolates in the SNP (orange), microsatellite (yellow), and DBLα type (green) “cleaned infections” datasets for the pre- **(C)** and post-IRS **(D)** surveys (**Table S2**). The median MOI*_var_* values for each marker are indicated with the black dashed lines.

**Figure 3:**
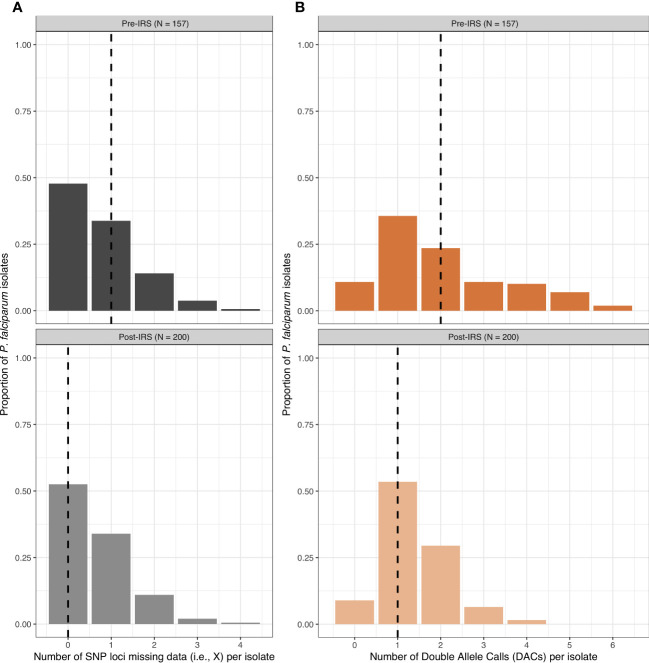
The frequency distributions for the number of SNP loci missing data (grey) **(A)** and the number of double-allele calls (DACs) (orange) **(B)** for the *P. falciparum* isolates included in the “cleaned infections” datasets pre- and post-IRS. The black dashed lines in each plot indicate the median number of SNP loci missing data per isolate (pre-IRS median = 1 [IQR: 0 – 1]; post-IRS median = 0 [IQR: 0 – 1]) and the median number DACs per isolate (pre-IRS median = 2 [IQR: 1 – 3]; post-IRS (median = 1 [IQR: 1 – 2]) are indicated with the black dashed lines (**Tables S4**, **S5**). For those isolates included in the pre- (N = 157) and post-IRS (N = 200) “cleaned infections” datasets for the SNPs, see **Data Sheets 1** and **2**, respectively.

From the 200 isolates selected pre- and post-IRS, SNP barcoding data was successfully obtained from 161 (80.5%) and 200 (100%) isolates in the pre- and post-IRS surveys, respectively. Using these successfully genotyped isolates, the SNP calls were then aggregated and all SNP loci with a call rate of at least 80% in both surveys were included (Daniels et al., 2008; Daniels et al., 2013; Daniels et al., 2015). Based on this call rate, four loci (i.e., A4, A10, B10, and B12) were removed, resulting in 20-SNP loci being used for the molecular analyses (**Table S3**). Finally, only those isolates with ambiguous or missing calls at four or fewer of the 20-SNP loci (≤ 20%) were included in downstream analyses (Daniels et al., 2008; Daniels et al., 2013; Daniels et al., 2015), resulting in 157 (78.5%) and 200 (100%) isolates in the pre- and post-IRS surveys, respectively (**Figures 2**, **3**). These isolates were defined as the “cleaned infections” dataset (**Figure 2**). All isolates included in the “cleaned infections” dataset were then evaluated to determine if they were monoclonal or multiclonal infections following the 24-SNP barcode data exclusion criteria of Daniels et al (Daniels et al., 2008; Daniels et al., 2013; Daniels et al., 2015). All isolates with more than one of the 20-SNP loci (> 5%) showing a double-allele call (DAC) were considered as multiclonal infections (i.e., MOI > 1) (**Figure S3**) and were excluded from the population genetic analyses (**Figure 3**, **Tables S4**, **S5**). We used this threshold to allow for the fact that one SNP may be miscalled as a DAC (i.e., low-level genotyping error) even in a monoclonal infection (Daniels et al., 2008; Nkhoma et al., 2013; Daniels et al., 2015). Based on this cut-off, only 73 (46.5%) and 125 (62.5%) isolates in the pre- and post-IRS surveys, respectively, were classified as monoclonal infections and included in the “monoclonal infections” dataset for analysis (**Figure 2**).

#### 3.1.2 Microsatellites

All details for the microsatellite genotyping and data cleaning for the 200 isolates selected pre- and post-IRS have been previously published (Argyropoulos et al., 2021). Briefly, using ten microsatellite loci, 192 (96.0%) and 200 (100%) isolates with genotyping data at ≥ 3 microsatellite loci in the pre- and post-IRS surveys, respectively, were included in the “cleaned infections” dataset (**Figure 2**). All isolates with (i) “true” monoclonal infections (i.e., one allele at all ten loci) or (ii) a maximum of two alleles at any locus (i.e., MOI = 2) where a dominant multilocushaplotype could be constructed (or phased) (Anderson et al., 2000; Ruybal-Pesántez et al., 2017a; Argyropoulos et al., 2021), were included in the “monoclonal infections” dataset, since phasing is possible for up to two microsatellite haplotypes. This resulted in 128 (66.7%) and 156 (78.0%) isolates from the pre- and post-IRS surveys, respectively (**Figure 2**).

#### 3.1.3 DBLα types

High-quality sequencing data (i.e., ≥ 20 DBLα types) was obtained from 172 (80.0%) and 193 (96.5%) isolates in the pre- and post-IRS surveys, respectively (i.e., “cleaned infections” dataset) (**Figure 2**; **Table S1**). Using *var*coding we determined the number of unique DBLα types in each isolate. Since the underlying population structure of the *var* multigene family in Bongo pre-IRS was characterized by non-overlapping DBLα isolate repertoires (Ruybal-Pesántez et al., 2022), we were able to use this limited similarity of repertoires in an isolate in high transmission to estimate clonality of infections (i.e., mono- or multiclonal). Therefore, in comparison to the SNPs and microsatellites, the *var*coding approach allowed for the inclusion of all isolates successfully genotyped for the data analyses, regardless of their MOI as phasing (i.e., haplotype construction) was unnecessary. As a result, all isolates successfully genotyped pre-IRS (N = 172) and post-IRS (N = 193) were included (**Figure 2**).

### 3.2 Genetic diversity

Even in isolates selected for low complexity, we found that 19 and 20 of the SNP loci were biallelic in the pre- and post-IRS surveys, respectively (**Tables S4**, **S5**), with the mean number of alleles per locus (*A*) being 1.9 and 2.0 in the pre- and post-IRS surveys, respectively (**Table 1**). All the microsatellite loci genotyped were polymorphic pre-IRS (from 5 to 20 alleles per locus) and post-IRS (from 5 to 22 alleles per locus) with the mean number of alleles per locus (*A)* being similar in both surveys, despite the IRS intervention (**Table 1**) (Argyropoulos et al., 2021). Using the DBLα types, we found that diversity, as measured using richness, was considerable, with 7,736 and 7,251 unique DBLα types being identified pre- and post-IRS, respectively (**Table 1**).

**Table 1:** Patterns of *P. falciparum* genetic diversity using the “monoclonal infections” datasets for the SNPs, microsatellites, and DBLα types pre- and post-IRS (**Figure 2**).

	Pre-IRS (October 2012)				Post-IRS (October 2015)			
Genetic markers	N	*h*	*A*	*H_e_*	N	*h*	*A*	*H_e_*
SNPs	73	71	1.9	0.25	125	106	2.0	0.24
Microsatellites	128	128	11.5	0.79	156	155	12.4	0.81
DBLα types	172	172	7,736 *	0.98 **	193	193	7,251 *	0.98**

N = number of isolates; *h* = number of unique haplotypes; *A* = mean number of alleles per locus (SNPs, microsatellites) (*) Note for DBLα types this number reflects the total number of unique DBLα types (i.e., richness); *H_e_* = expected heterozygosity (**) Note for the DBLα types the expected heterozygosity was measured using *var* expected heterozygosity (*H_v_*) as described in the Materials and Methods.

For the pre-IRS survey, we found the number of haplotypes (*h*) matched the number of isolates (N) at each marker, except for the SNPs where two isolates shared the same infection haplotype (**Table 1**). We found that all haplotypes in the post-IRS survey were unique for the microsatellites (except for two participants that shared the same haplotype) (Argyropoulos et al., 2021) and the DBLα types. Conversely, using the biallelic SNPs only 106 unique multilocus haplotypes were observed from the 125 isolates, indicating that there were repeated 20-SNP barcodes in the population, thereby underestimating the genome diversity compared to the microsatellites and DBLα types. Finally, *H_e_* (*H_v_* for the DBLα types) remained relatively stable pre- to post-IRS across all three genetic markers (**Table 1**). However, *H_e_* based on the SNPs was lower than those based on the microsatellites and DBLα types for both surveys (i.e., pre-IRS and post-IRS). Thus, by using less diverse markers that require the selection of monoclonal infections (or those with the lowest complexity) we were underestimating parasite diversity in this high-transmission setting in SSA.

### 3.3 Genetic similarity

To undertake the comparisons for the SNPs and microsatellites using the P_AS_ statistic, we only used isolates with “monoclonal infections” and complete multilocus infection haplotypes (i.e., no missing genotype data) resulting in 34 (21.7%) and 68 (34.0%) isolates for the pre- and post-IRS SNPs, and 81 (42.2%) and 84 (42.0%) isolates for the pre- and post-IRS microsatellites (**Figure 2**). The advantage of using DBLα types and the PTS statistic to assess genetic similarity, compared to the SNPs and microsatellites, is that we can compare all isolates genotyped irrespective of their MOI*_var_* (i.e., repertoire size) or missing data because phasing is not necessary. Thus, all of the isolates successfully *var*coded during the pre- (N = 172) and post-IRS (N = 193) surveys were included for this analysis (**Figure 2**).

**Figure 4:**
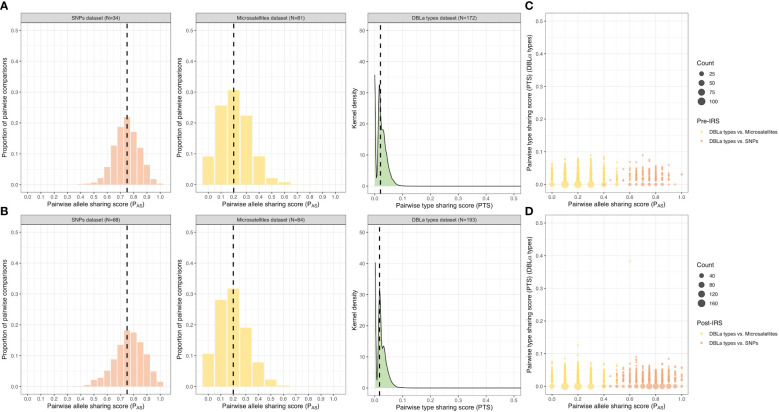
Genetic similarity among the pre- and post-IRS surveys. Distribution of the pairwise allele sharing (P_AS_; SNPs and microsatellites) and pairwise type sharing (PTS; DBLα types) scores and comparisons. Genetic similarity of the *P. falciparum* isolates in the “monoclonal infections” with complete haplotypes datasets (i.e., no missing genotype data) pre- **(A)** and post-IRS **(B)** for the SNPs (orange), microsatellites (yellow), and DBLα types (green) (**Figure 2**). The median P_AS_ and PTS scores are indicated with black dashed lines (please see **Table S6** for more details). Both P_AS_ and PTS were used as a similarity or relatedness statistic: where “0 - 0.50” = dissimilar or unrelated, “0.5” = recent recombinants/siblings, “> 0.5” = similar or related, and “1” = clones/identical. Pairwise genetic similarity comparisons (PTS versus P_AS_) pre- **(C)** and post-IRS **(D)**. Points represent the isolate genetic similarity comparisons for the DBLα types versus SNPs (orange; pre-IRS N = 32 and post-IRS N = 65 isolates compared) and DBLα types versus microsatellites (yellow; pre-IRS N = 77 and post-IRS N = 83 isolates compared). Note that the x-axis and y-axis scales are different, ranging from 0 – 0.5 and 0 – 1 for the PTS and P_AS_ scales, respectively. For the genetic similarity comparisons for the microsatellites versus SNPs see **Figure S4**.

Using the SNP P_AS_ comparisons, we observed that the majority of isolate infection haplotypes in both the pre- and post-IRS surveys were highly similar as they shared ≥ 0.75 alleles in their SNP barcodes (i.e., identical ≥ 15 out of the 20 loci genotyped) (**Figures 4A, B**; **Table S6**). Although the median P_AS_ score was the same in both surveys, the P_AS_ distributions were significantly different with a shift towards higher P_AS_ values post-IRS compared to pre-IRS (*p-value* < 0.001, Mann Whitney U test) (**Figures 4A, B**; **Table S6**). This result based on SNPs implies that isolate haplotypes in the population became more similar (i.e., increased sharing of alleles) following the IRS intervention. Although these high P_AS_ scores indicate that these isolates are highly similar and could be related, this must be interpreted with caution since P_AS_ is sensitive to the local minor allele frequencies (MAF) of the SNP panel and thus higher P_AS_ scores may be observed when a greater number of low-heterozygosity loci are included (Speed and Balding, 2015; Taylor et al., 2019). Since ~45-50% of the loci had MAF ≤ 0.1 (10%) (i.e., one highly dominant allele at these loci) (**Tables S4**, **S5**), this 20-SNP barcode is expected to be less informative for assessing genetic similarity in this population compared to the more polymorphic molecular markers such as the microsatellites and DBLα types (**Figures 4C, D**, **S4**).

When we evaluated similarity using the multiallelic microsatellites pre- and post-IRS data, the majority of isolate infection haplotypes were found to be dissimilar or unrelated as they only shared ≤ 0.2 of their alleles (i.e., identical at two or fewer loci out of ten) (**Figures 4A, B**; **Table S6**), making them more informative than SNPs to determine genetic similarity in this population. P_AS_ distributions using microsatellites showed that isolates were significantly less similar (i.e., lower P_AS_ values) post-IRS than pre-IRS (*p-value* < 0.001, Mann Whitney U test) despite the median P_AS_ scores being the same (**Figures 4A, B**; **Table S6**). Finally using the DBLα type data, we found that 99.9% of the PTS comparisons were ≤ 0.1 (i.e., shared ≤ 10% of their DBLα types), indicating that DBLα isolate repertoires in this population were highly dissimilar and composed of diverse DBLα types both pre- and post-IRS (**Figures 4A, B**; **Table S5**). Using this analysis, we found that the PTS distributions were significantly different (*p-value* < 0.001, Mann Whitney U test) and that there was a shift towards a lower median PTS value post-IRS (i.e., less similar) (**Figures 4A, B**; **Table S6**).

Since DBLα types were the most diverse marker, they provided higher resolution to distinguish between similar and dissimilar infection haplotypes compared to the biallelic SNPs and multiallelic microsatellites (**Figures 4C, D**, **S4**). In fact, when we compared the same set of isolates with both P_AS_ (SNPs or microsatellites) and PTS scores, we observed that isolates that were identical or highly similar using their SNP barcodes, were found to be dissimilar (or unrelated) when assessed using their DBLα isolate repertoires (**Figures 4C, D**).

### 3.4 *Var*coding analyses for all infections

Given that the *var*coding approach allows for the analysis of all isolates regardless of clonality, we were able to further analyze the population structure of all isolates successfully *var*coded in the larger initial dataset pre- (84.8%, N = 685) and post-IRS (75.8%, N = 413) (**Table S1**) without the need to subsample for low-complexity infections. Using this larger dataset, we found that the parasite reservoir in Bongo was still composed of highly dissimilar DBLα isolate repertoires (median PTS [IQR]: pre-IRS = 0.033 [0.021-0.046] and post-IRS = 0.023 [0.013 - 0.035]) that became less similar following the IRS intervention (*p-value* < 0.001, Mann Whitney U test). Thus, the reduced similarity reported using the sample of low-complexity infections in this analysis was confirmed with observations based on the larger dataset.

**Figure 5:**
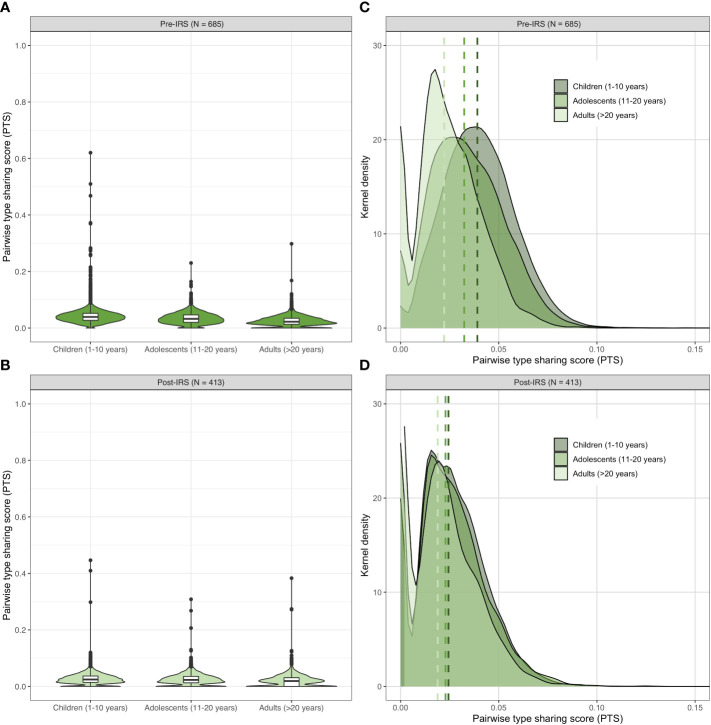
Age-specific patterns of genetic similarity in the pre- and post-IRS surveys. Violin plots showing the pairwise type sharing (PTS) distributions of all *P. falciparum* isolates with DBLα sequence data (**Table S1**) in each age group pre- **(A)** and post-IRS **(B)**. The box plots for each age group show the median and interquartile ranges and the black dots denote outliers. Kernel density plots showing the lower end of the PTS distributions for each age group pre- **(C)** and post-IRS **(D)**. The PTS scales in the density plots have been zoomed-in to provide better visualization of the DBLα types PTS distributions. The dashed lines indicate the median PTS values for the children (1-10 years, dark green), adolescents (11-20 years, medium green), and adults (> 20 years, light green) (please see **Table S7** for more details).

As we were able to *var*code all isolates in the larger dataset, additional analyses of population structure by age (i.e., children: 1–10 years, adolescents: 11-20 years, and adults: > 20 years) were possible. Although multiclonal infections were observed across all age groups in Bongo, children (1–10 years) and adolescents (11-20 years) carried the largest proportion of these multiclonal infections pre- and post-IRS, as previously published (Tiedje et al., 2022). Using these age stratifications, we found that not only was there limited overlap of DBLα isolate repertoires in each age group both pre- and post-IRS (**Figure 5**; **Table S7**) but that repertoire similarity was significantly higher in the children compared with the adolescents and adults pre-IRS (for further discussion see Ruybal-Pesántez et al. (2022)). This age-specific pattern was maintained post-IRS (*p-value* < 0.001 for all comparisons, Mann Whitney U test). Finally, by examining the PTS distributions, we observed that DBLα isolate repertoires were significantly less similar in each age group pre- to post-IRS (*p-value* < 0.001 for all comparisons, Mann Whitney U test) (**Figure 5**; **Table S7**). Again, this confirmed the detection of the subtle but significant decrease in similarity following the IRS intervention. This result shows that we can get a snapshot of changes in population structure due to the intervention by *var*coding with samples taken from any of these three age classes without the need to limit analyses to only monoclonal infections.

## 4 Discussion

Our study highlights the challenge of doing *P. falciparum* population genetics in the highest burden countries of SSA where ~550 million people live at risk of infection (World Health Organization & Roll Back Malaria Partnership to End Malaria, 2019; World Health Organization, 2021). We have addressed several interconnected issues related to defining appropriate methods for molecular surveillance of *P. falciparum* using low-density asymptomatic infections to capture changes in population genetics at local or regional levels as a result of vector control interventions. Namely, the importance that marker choice has in relation to prevalence of multiclonal infections to resolve diversity and population structure estimates in relatively small sample sizes. When assessing the impact of an IRS intervention by three different markers, the resolution of population genetics estimates increased with marker polymorphism. The extent of multiclonal infections was particularly an issue related to data availability for biallelic SNP and microsatellite analyses due to phasing issues. In contrast, *var*coding worked relatively independent of MOI in high transmission (Day et al., 2017; Ruybal-Pesántez et al., 2017b; Ruybal-Pesántez et al., 2022) and provided informative data with relatively small sample sizes. Microsatellite genotyping provided similar population structure data, but required the selection of low-complexity infections from the larger initial sample.

An important result of our study is the failure to increase *var* repertoire or microsatellite haplotype similarity (or relatedness) by the IRS intervention. We start our intervention with very high *var* repertoire diversity in the asymptomatic parasite population. We show IRS reduced transmission intensity by > 90% and so we expect to have greatly reduced outcrossing by both reducing the population of biting mosquitos and the lifespan of blood fed mosquitos thereby minimizing exposure of humans to new infections. As a consequence, we are largely looking at the decay of the parasite population in the human host (asymptomatic reservoir) with few new transmission events. As this parasite population at baseline pre-IRS has low *var* similarity, it maintains this feature or becomes less similar as the number of *var* repertoires to be compared declines post-IRS. Microsatellite genotyping shows the same pattern of low similarity for genome diversity per se, pre- and post-IRS. The extent of diversity post-IRS may also be contributed to via migration of diverse parasites from uncontrolled areas as our study site shares an immediate border with Burkina Faso. Some evidence for such migration came from our earlier microsatellite work where we observed significant spatiotemporal differentiation in Bongo. In this analysis we showed that not only were the catchment areas (i.e., Vea/Gowrie and Soe) genetically different post-IRS, but that the parasite population in Soe (proximal to Burkina Faso) was genetically differentiated pre- to post-IRS as measured using Jost’s *D* and *G_ST_* (Argyropoulos et al., 2021).

A study from Thiès, Senegal a peri-urban region, showed the opposite result where they saw a shift towards more similarity and clonality following interventions targeting clinical infections (i.e., rapid diagnostic tests and artemisinin-based combination therapies) and transmission intensity (i.e., LLINs and IRS) simultaneously (Daniels et al., 2015). So, what is different about the Thiès, Senegal and Bongo, Ghana studies with opposite outcomes? We point to the overall diversity of the parasite populations in low vs high transmission. Firstly Thiès, prior to intervention scale-up was a low-transmission area as defined by the WHO (i.e., prevalence ≤ 10% and an annual incidence of 100-250 cases/1000) (Mouzin et al., 2010; Daniels et al., 2015; WHO/GMP, 2017). Whereas Bongo remained classified as a high-transmission area pre- and post-IRS (see Materials and Methods) maintaining a very large reservoir of asymptomatic infections with a similar incidence of clinical cases to Thiès in 2006 (Ghana Health Service; Tiedje et al., 2017; Tiedje et al., 2022). Secondly in Thiès, they sampled and analyzed clinical cases of malaria and a saw a > 95% reduction between 2006 and 2009. While in Bongo we undertook longitudinal sampling of the asymptomatic reservoir across all ages in a ~60 km^2^ area and saw a 37.5% decline in *P. falciparum* prevalence (Tiedje et al., 2022). Thus, the size of the parasite populations both pre- and post-intervention differs substantially in these studies. From population genetic theory, small amounts of outcrossing will have a greater impact in increasing parasite similarity (or relatedness) in a small parasite population as seen in low transmission.

By measuring the genetic diversity of loci, haplotypes, and P_AS_/PTS for three established molecular assays, we get different pictures of both the extent of genetic diversity and similarity of the *P. falciparum* reservoir in Bongo pre- and post-IRS. Given the same initial sample size of ~200 isolates with low-complexity infections, the variable resolution of the methods relates to the relative polymorphism of the markers (i.e., SNPs < microsatellites < DBLα types). Biallelic SNPs showed a less diverse parasite reservoir both pre- and post-IRS, underestimating the greater diversity of genomes seen with microsatellites and *var*coding. The reduced diversity and higher genetic similarity observed using the SNP barcode was largely due to the biallelic nature of SNPs and the necessary removal of multiclonal infections, reducing sample size for analysis. Multiallelic microsatellites showed a parasite reservoir that was diverse and genetically dissimilar during both the pre- and post-IRS surveys. When considering measuring changes in neutral variation, it is clear that the more polymorphic microsatellite markers have greater resolution than the 20-SNP barcode. Genotyping a larger panel of SNP or microsatellite markers (e.g., ≥ 200 biallelic or 100 polymorphic loci to achieve low error rates) to undertake relatedness estimates using IBD has been recommended (Taylor et al., 2019; Han et al., 2022), but the same problems will occur as the number of markers is not the issue in high-transmission areas. Instead, it is the relative polymorphism of the marker.

DBLα types showed that the parasite isolates were highly diverse and composed of dissimilar *var* repertoires pre- and post-IRS. In fact, the lack of the need for phasing with DBLα types provided more usable data for *var*coding than the SNP or microsatellite markers. Most significantly, *var*coding picked up a subtle change in *var* repertoire similarity of the parasite population post-IRS. As the IRS intervention reduced transmission intensity, it makes sense that isolate *var* repertoire similarity would reduce as a consequence of less outcrossing in the mosquito, limiting the possibility to create more similar or related recombinant genomes. *Var*coding was able to detect this change in any age group and any infection complexity in sample sizes of up to 200 isolates. This observed reduction in similarity post-IRS was further confirmed by the analysis of all data from *var*coding of microscopy-positive infections sampled from the larger cohort of ~2,000 participants (see **Figure 5** and **Table S7**) as all of the high MOI*_var_* data were usable.

Cost estimates of molecular genotyping assays for surveillance usually focus on the price of reagents per sample, but the sample size required to get informative population genetic data with an assay also contributes significantly to dollars spent. This can vary tenfold as shown in this study where pre-screening ~2,000 participants of all ages had to be performed to successfully identify less than 200 monoclonal infections per survey suitable for SNP barcoding and microsatellite genotyping. Isolates collected from residents with low-density asymptomatic infections were genotyped in this study, resulting in isolates being excluded due to low-quality genotyping data or missing data in the “cleaned infections” datasets for all three markers analyzed. While a pre-amplification step with selective whole-genome amplification (sWGA) is required by other panels to improve performance when amplifying DNA from low-density infections from dried blood spots (e.g., 10 parasites/μl of blood), it adds a considerable cost to the genotyping assays (Oyola et al., 2016; Jacob et al., 2021; LaVerriere et al., 2022). Although sWGA could increase the number of isolates available to assess diversity and genetic relatedness for all markers, it does not resolve the issue of multiclonal infections; this is what actually limits the use of SNPs in high transmission due to their biallelic nature and the high proportion of DACs, leading to reduced numbers of usable multilocus haplotypes (**Figure S3**).

More recently with the development of newer genetic panels composed of a larger number of biallelic SNPs (Jacob et al., 2021; LaVerriere et al., 2022) or multiallelic microhaplotypes (Tessema et al., 2020) (≥ 2 SNPs within a DNA segment unbroken by recombination (Baetscher et al., 2018)), statistical packages (e.g., DEploid (Zhu et al., 2018), DEploidIBD (Zhu et al., 2019), and Dcifer (Gerlovina et al., 2022)) have been developed using identity-by-decent (IBD) based methods to estimate relatedness for phased or unphased multiclonal infections. Although promising, they have been developed on limited datasets from high transmission showing greater error for isolates with greater than five clones and are yet to be tested in the field. Such methods do not consider the extent of outcrossing in natural populations, especially in high transmission (Babiker et al., 1994; Paul and Day, 1998), where haplotypes are not stable in epidemiologic time.

Besides the markers compared in this study, additional genetic panels have been developed for molecular surveillance of malaria parasites in the field. These newer panels, including SpotMalaria v2 (Jacob et al., 2021), Paragon v1 (Tessema et al., 2020), and AMPLseq v1 (LaVerriere et al., 2022), have been designed for multiplexed PCR amplicon sequencing and incorporate single copy antigenic loci under selection, known antimalarial drug resistance markers, biallelic SNP loci, and/or microhaplotypes. *In silico* validation of these panels from countries with low or high parasite diversity have shown they are more accurate to assess genetic relatedness compared to the 24-SNP barcode. Nonetheless, simulated monoclonal infections were needed for this analysis using AMPLseq v1 (LaVerriere et al., 2022). Until there is sufficient local population genetic data to train computational approaches to accurately phase in the MOI ranges typical of high-transmission settings, even these newer panels with deeper coverage at a larger number of loci (i.e., > 100 SNPs and/or microhaplotypes) are not sufficient to overcome the issue of phasing of multiclonal infections.

If the primary goal for these molecular surveillance methods is to be used in countries to directly inform National Malaria Control/Elimination Programmes, cost-effective and scalable platforms will be necessary. Although microsatellites have been informative in a variety of malaria transmission settings, they have unfortunately proven to be technically difficult to standardize across laboratories due to issues with allele calling and errors in the assessment of clonality (Figan et al., 2018). New platforms are emerging to use these markers which should reduce cost and be easier to use (Han et al., 2022). The advantages of *var*coding and the newer panels (i.e., SpotMalaria v2, Paragon v1, and AMPLseq v1), using amplicon sequencing, is that large number of isolates can be multiplexed into a single-pool, thus overall costs are mainly driven by the number of PCRs/clean-up, the number of samples indexed per sequencing run, and the next-generation sequencing (NGS) technology used ($20 to $40 USD per isolate) (Tessema et al., 2020; LaVerriere et al., 2022). However, several key features set *var*coding apart from these other methods. First, *var*coding can amplify DNA collected as dried blood spots and stored for more than 5-years from both clinical or low-density asymptomatic infections (Day et al., 2017; Ruybal-Pesántez et al., 2017b; Ruybal-Pesántez et al., 2021; Ruybal-Pesántez et al., 2022), without the added cost and additional step of sWGA ($8 to $32 USD per isolate, for costing estimates see (Tessema et al., 2020; LaVerriere et al., 2022). Second, since all *P. falciparum* genomes possess ~40 to 60 *var* genes (Bull et al., 1998; Gardner et al., 2002; Rask et al., 2010; Otto et al., 2019) and there is extensive repertoire diversity (Day et al., 2017; Ruybal-Pesántez et al., 2017b; He et al., 2018; Ruybal-Pesántez et al., 2022), *var*coding can be used for molecular surveillance both locally and globally without the need to be customized or updated (Chen et al., 2011; Day et al., 2017; Rougeron et al., 2017; Ruybal-Pesántez et al., 2021; Tonkin-Hill et al., 2021; Ruybal-Pesántez et al., 2022). Thus, with a simple PCR using degenerate primers, *var*coding can be easily deployed in malaria endemic regions (Tonkin-Hill et al., 2021).

In conclusion, we have exploited the fact that *var* genes and *var* repertoires diversify by recombination to create *var*coding. Here we show that this method can be used in high-transmission settings to measure diversity and population structure even in multiclonal infections. This is achieved more easily than SNP barcoding and microsatellite genotyping as there is no need for pre-selection of isolates and phasing. Measuring PTS or IBS complements other previously published uses of the method to measure MOI (Ruybal-Pesántez et al., 2022; Tiedje et al., 2022; Labbé et al., 2023) as well as identify geographic signatures at the country level within Africa to assess importation of parasite *var*codes (Ruybal-Pesántez et al., 2017b; Tonkin-Hill et al., 2021; Ruybal-Pesántez et al., 2022). These three measurements can be made robustly in relatively small sample sizes. This triple output of the method was not seen with either SNP barcoding or microsatellite genotyping.

## Data availability statement

The SNP and microsatellite datasets used for this analysis are available in Dryad at https://doi.org/10.5061/dryad.jsxksn0bp and https://doi.org/10.5061/dryad.kh189324z, respectively. The DBLα sequences utilized in this study are publicly available in GenBank (https://www.ncbi.nlm.nih.gov/genbank/) under BioProject Number: PRJNA 396962. All custom code is available in an open source repository: (i) DBLαCleaner pipeline is available at https://github.com/UniMelb-Day-Lab/DBLaCleaner, (ii) clusterDBLalpha pipeline is available at https://github.com/Unimelb-Day-Lab/clusterDBLalpha, and the (iii) classifyDBLalpha pipeline is available at https://github.com/Unimelb-Day-Lab/classifyDBLalpha. A tutorial and dataset to demo this custom code is available at https://github.com/UniMelb-Day-Lab/tutorialDBLalpha.

## Ethics statement

The studies involving human participants were reviewed and approved by Navrongo Health Research Centre, Noguchi Memorial Institute for Medical Research, The University of Chicago, and The University of Melbourne. Written informed consent to participate in this study was provided by the participants’ legal guardian/next of kin.

## Author contributions

KPD, KAK, MP conceived and designed the study. AG, KET, DCA, COO, SLD processed the samples and performed the genotyping experiments. AG, KET, DCA processed, cleaned, and curated the datasets for analysis. AG, KET, DCA, FL analyzed the data. KET, DCA visualized the data. KPD, KET, DCA wrote the original draft of the manuscript. AG, COO, SLD, FL, ARO, KAK, MP reviewed and edited manuscript. KPD, ARO, KAK supervised the research. ARO, KAK, MP, KPD acquired the funding. All authors contributed to the article and approved the submitted version.
