# Supplementary material for: Comparison of molecular surveillance methods to assess changes in the population genetics of Plasmodium falciparum in high transmission
Source: Front Parasitol. Author manuscript; Available in PMC 2023 Nov 29. (PMC10686283; doi:10.3389/fpara.2023.1067966)
Supplement: Supplemental Material [file NIHMS1892206-supplement-Supplemental_Material.pdf]

# *Supplementary Material*

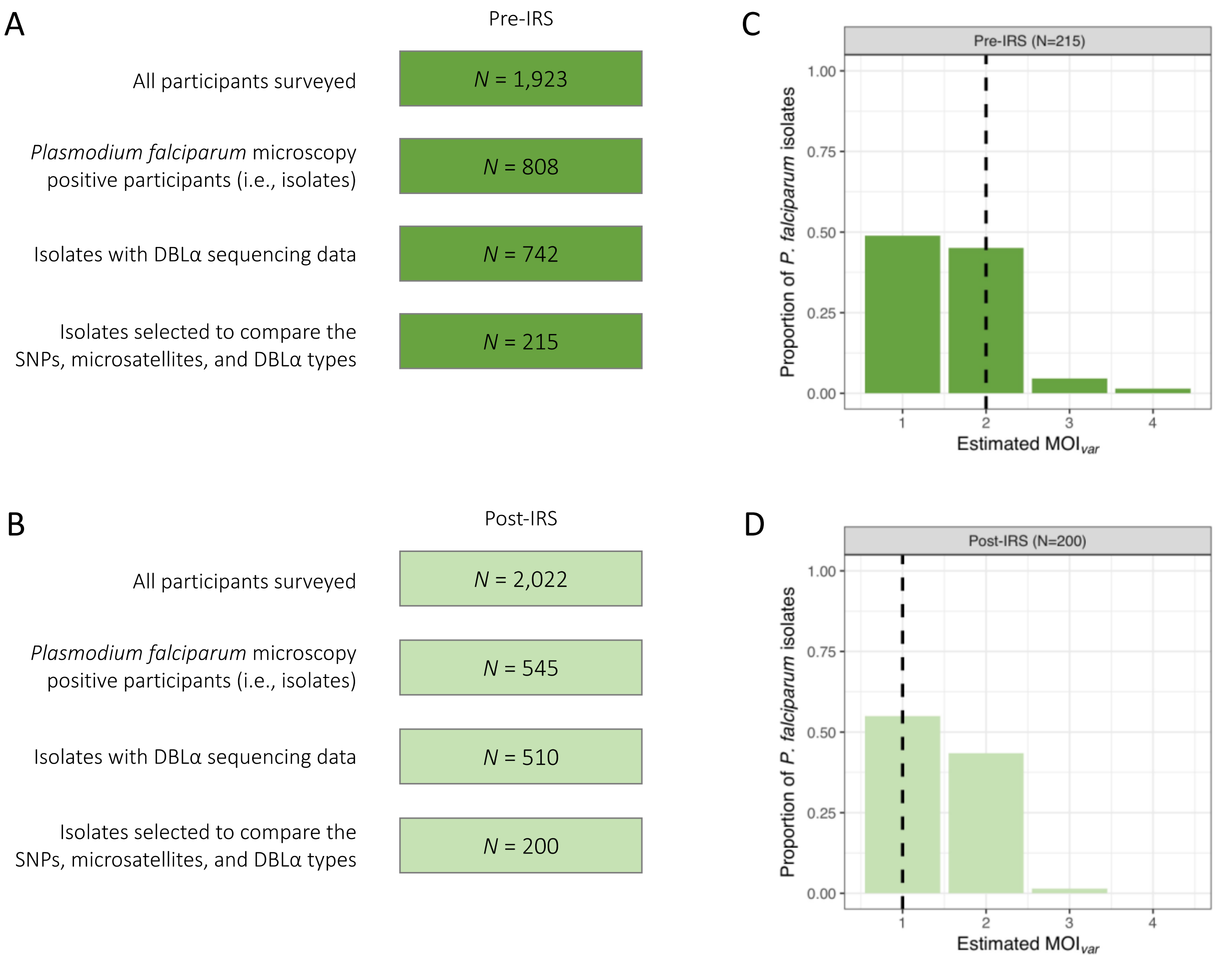


**Figure S1. Pre- and post-IRS data for those *P. falciparum* isolates selected for the population genetic analyses.** Breakdown of the participants surveyed and the *P. falciparum* isolates selected with the lowest complexity of infection and DBLα sequencing data in the pre- (**A**) and post-IRS (**B**) surveys (Table S1). $MOI_{var}$ frequency distributions of the *P. falciparum* isolates selected for genotyping in the pre-IRS (**C**, N = 215; median $MOI_{var}$ = 2 [IQR: 1 – 2] indicated with the black dashed line) and post-IRS (**D**, N = 200; median $MOI_{var}$ = 1 [IQR: 1 – 2] indicated with the black dashed line) surveys (Table S2). Note the isolates selected pre- and post-IRS were not statistically different than those isolates in the original study population for any of the key variables, except age pre-IRS (*p-value* < 0.001, Chi-square test) and parasitemia post-IRS (*p-value* < 0.01, Mann Whitney U test) (1).

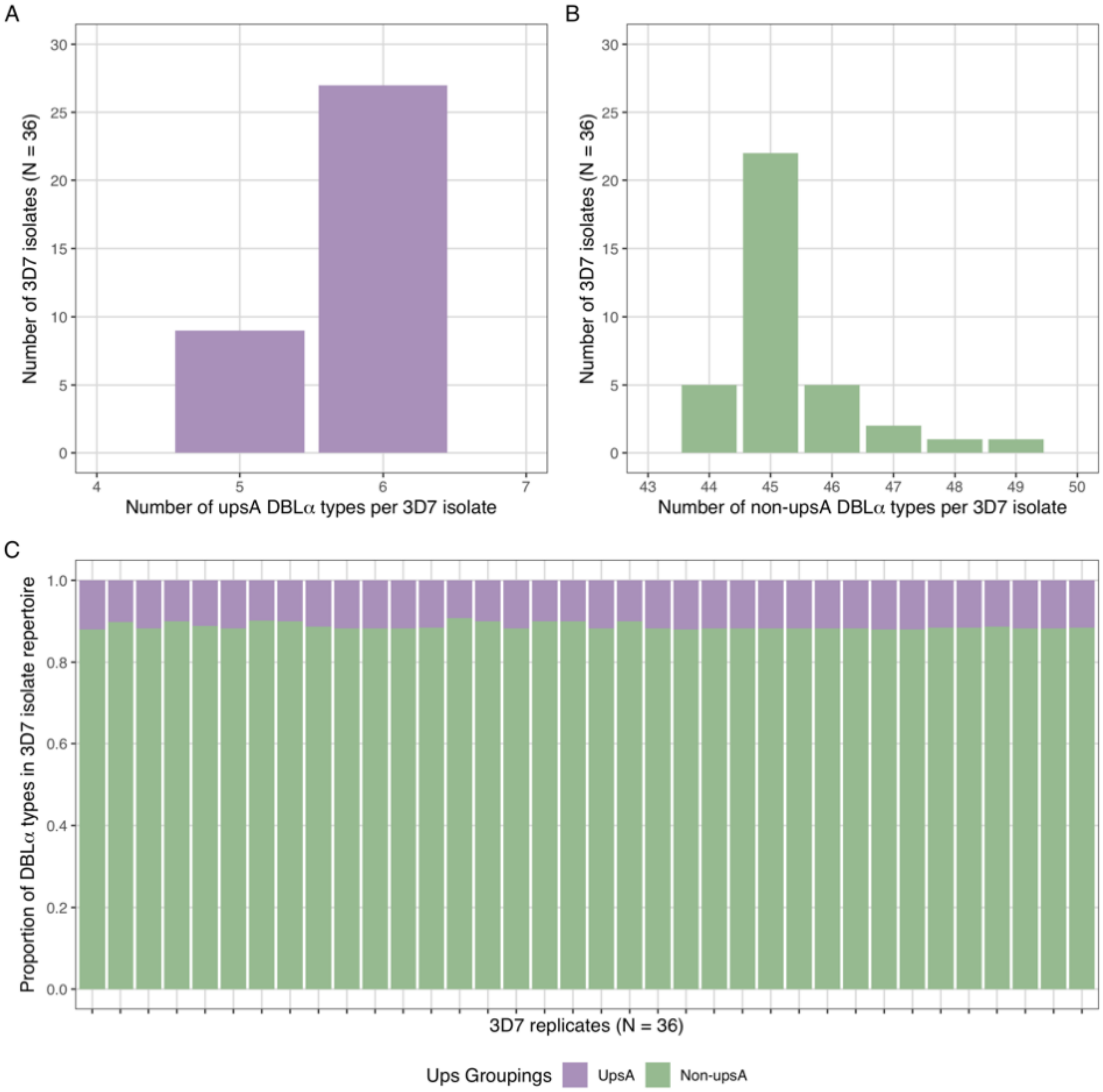


**Figure S2. UpsA (purple) and non-upsA (green) DBLα type data obtained from the 3D7 laboratory isolate included as a *P. falciparum* control (i.e., technical replicate, N = 36) during *var*coding.** The distribution of the number of upsA (**A**) and non-upsA (**B**) DBLα types identified in each of the 3D7 isolate repertoires (i.e., technical replicates, N = 36). From the data obtained we identified the expected repertoire sizes with a median of 6 upsA DBLα types per isolate (range: 5 - 6) and a median of 45 non-upsA DBLα types per isolate (range: 44 - 49). (**C**) The proportion of upsA and non-upsA DBLα types identified in each of the 3D7 isolate repertoires (i.e., technical replicates, N = 36). The median genomic proportions for the upsA DBLα types was 11.8% (range: 9.3-12.0%) and for the non-upsA DBLα types was 88.2% (range: 88.0-88.7%). These findings correspond to what was expected from whole genome sequencing data for 3D7 (2).

A

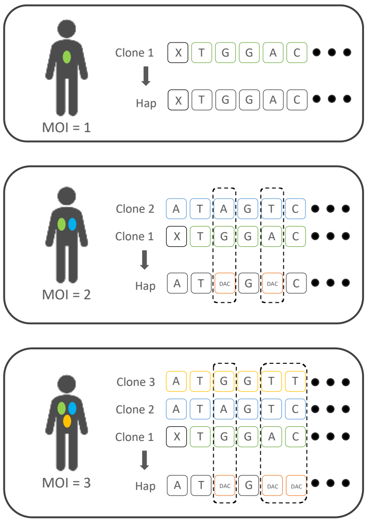


B

| SNP ID # | Pf_01_000130573 | Pf_01_000539044 | Pf_02_000842803 | Pf_04_000282592 | Pf_05_000931601 | Pf_06_000145472 | Pf_07_000277104 | Pf_07_000490877 | Pf_07_000545046 | Pf_07_000657939 | Pf_07_000671839 | Pf_07_000683772 | Pf_07_000792356 | Pf_07_001415182 | Pf_08_000613716 | Pf_09_000634010 | Pf_10_000082376 | Pf_11_000406215 | Pf_13_000158614 | Pf_13_001429265 | | |
|---|---|---|---|---|---|---|---|---|---|---|---|---|---|---|---|---|---|---|---|---|---|---|
| **Major allele** | **C** | **A** | **C** | **C** | **C** | **C** | **A** | **A** | **T** | **T** | **G** | **C** | **A** | **C** | **C** | **C** | **T** | **C** | **T** | **G** | | |
| **Minor allele** | **T** | **G** | **T** | **T** | **G** | **G** | **G** | **T** | **C** | **C** | **A** | **T** | | **A** | **A** | **A** | **A** | **A** | **C** | **T** | | |
| **Isolate ID** | **A1** | **B1** | **A2** | **B2** | **A3** | **B3** | **B4** | **A5** | **B5** | **A6** | **B6** | **A7** | **B7** | **A8** | **B8** | **A9** | **B9** | **A11** | **B11** | **A12** | **# DACs** | **Call** |
| 340 | C | A | DAC | C | C | C | DAC | A | T | DAC | DAC | DAC | A | C | C | DAC | T | C | T | G | 6 | multi |
| 401 | T | A | DAC | T | C | C | DAC | DAC | T | DAC | DAC | DAC | A | C | C | C | T | C | C | G | 6 | multi |
| 354 | C | A | DAC | T | C | C | DAC | A | T | T | DAC | DAC | A | C | DAC | A | T | C | T | G | 5 | multi |
| 405 | C | G | C | DAC | C | G | DAC | A | T | DAC | DAC | DAC | A | C | C | X | T | C | T | G | 5 | multi |
| 880 | C | A | C | C | C | C | DAC | A | T | T | DAC | DAC | A | C | DAC | DAC | T | C | T | G | 5 | multi |
| 1799 | C | G | X | C | C | C | G | DAC | T | DAC | DAC | DAC | A | C | C | X | T | A | T | G | 4 | multi |
| 32 | T | A | X | C | C | C | DAC | A | T | T | DAC | DAC | A | C | C | C | T | C | T | G | 3 | multi |
| 1879 | T | A | X | C | C | C | DAC | A | T | DAC | A | T | A | C | C | C | T | C | T | G | 2 | multi |
| 1137 | C | A | C | T | C | C | G | A | T | T | G | DAC | A | C | C | C | T | C | C | G | 1 | *mono* |
| 1177 | T | G | C | C | C | C | G | A | T | DAC | G | C | A | C | C | X | T | C | T | G | 1 | *mono* |
| 1920 | T | A | T | C | C | C | A | T | T | C | G | C | A | C | A | A | A | C | T | G | 0 | *mono* |
| 1998 | C | X | C | C | C | C | A | T | T | T | G | C | A | C | A | C | T | C | C | G | 0 | *mono* |

**Figure S3. An illustrative example of the process of constructing (i.e., phasing) the 20-SNP barcode isolate haplotypes. (A)** Schematic diagram depicting the phasing of monoclonal infections (MOI = 1) versus the difficulties in phasing multiclonal infections (MOI > 1) to construct haplotypes (Hap). Those loci with double-allele calls (DACs) are shaded in orange, while those with ambiguous or missing allele calls (X) are shaded in dark grey. **(B)** To illustrate the difficulties in phasing multiclonal infections, we have provided SNP barcodes from 12 isolates in the pre-IRS survey (DACs range: 1-6). SNP calls are shown for the 20-loci included in this analysis (SNP ID #) (DACs, orange; ambiguous or missing allele calls (X), dark grey). Isolates with ≤ 1 DAC are considered monoclonal infections (i.e., Call = "*mono*"), while those with ≥ 2 DACs are considered multiclonal infections (i.e., Call = "multi"). SNP barcodes are generated by genotyping biallelic SNP loci located across the *P. falciparum* genome, to construct multilocus haplotypes (3). If isolates are multiclonal, a substantial number of the SNP loci will be DACs, rendering the isolate data unsuitable for population genetic analyses due to the issues of phasing the multilocus haplotypes of mixed clone infections (4,5). Hence, only monoclonal infections can be used to construct multilocus haplotypes, as reported by Daniels et al. (2015)(5). See Data Sheet 1 and Data Sheet 2 for the 20-SNP barcodes for all isolates pre-IRS (N = 157) and post-IRS (N = 200), respectively.

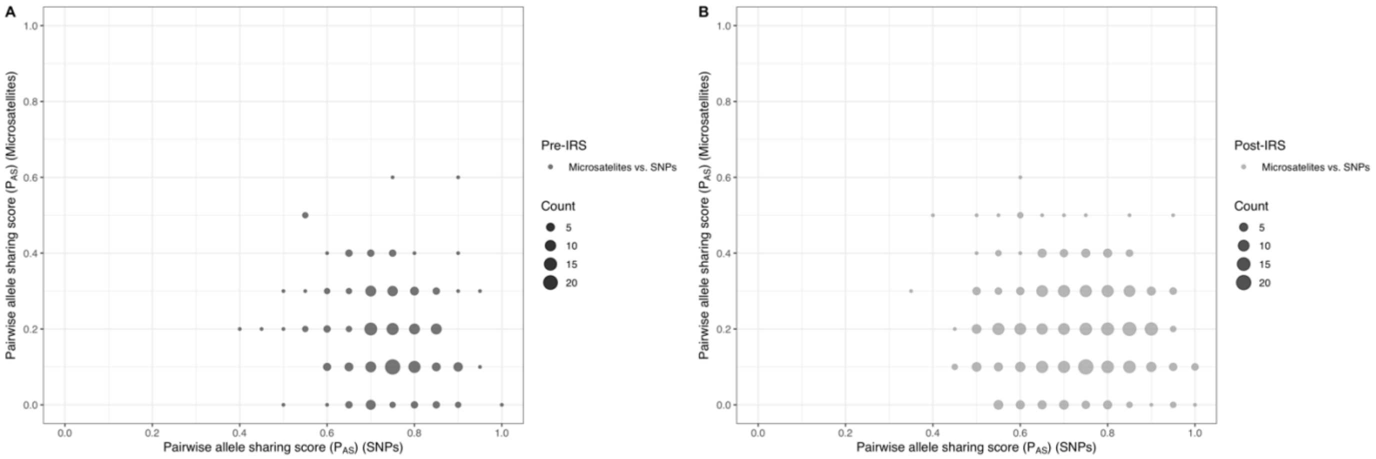


**Figure S4. Pairwise genetic similarity comparisons.** Points represent the pairwise allele sharing ($P_{AS}$) comparisons for the microsatellites versus SNPs for the pre- (**A**; dark grey, N = 20 isolates compared) and post-IRS (**B**; light grey, N = 27 isolates compared) surveys.

**Table S1.** DBLα sequencing results for all isolates that were positive for an asymptomatic *P. falciparum* infection by microscopy.

| Survey | Microscopic *P. falciparum* positive isolates sequenced | *P. falciparum* isolates with high-quality DBLα sequencing data (i.e., ≥ 20 DBLα types) [a] | *P. falciparum* isolates with limited DBLα sequencing data (i.e., 1-19 DBLα types) [b] | *P. falciparum* isolates with no DBLα sequencing data (i.e., 0 DBLα types) [c] |
|---|---|---|---|---|
| **Pre-IRS (October 2012)** | 808 | 685 (84.8) | 57 (7.0) | 66 (8.2) |
| **Post-IRS (October 2015)** | 545 | 413 (75.8) | 97 (17.8) | 35 (6.4) |

[a] Data reflect the number (% (n/N)) of microscopic *P. falciparum* isolates that had between DBLα sequencing data (i.e., ≥ 20 DBLα types) relative to the number of participants sampled that were microscopically positive for *P. falciparum* (including mixed *P. falciparum* infections).
[b] Data reflect the number (% (n/N)) of microscopic *P. falciparum* isolates that had between DBLα sequencing data (i.e., 1-19 DBLα types) relative to the number of participants sampled that were microscopically positive for *P. falciparum* (including mixed *P. falciparum* infections).
[c] Data reflect the number (% (n/N)) of microscopic *P. falciparum* isolates that had no DBLα sequencing data relative to the number of participants sampled that were microscopically positive for *P. falciparum* (including mixed *P. falciparum* infections).

**Table S2.** Demographic and parasitological characteristics of the *P. falciparum* isolates selected and those successfully genotyped (i.e., "cleaned infections" dataset) in the pre-IRS (October 2012) and post-IRS (October 2015) surveys. Note there were no statistical differences between those isolates successfully genotyped (i.e., "cleaned infections" dataset) pre- and post-IRS for the SNPs, microsatellites, and DBLα types and those isolates selected, except for parasitemia pre-IRS for the DBLα types (*p-value* < 0.01, Mann Whitney U test).

| | **Pre-IRS (October 2012)** | | | | **Post-IRS (October 2015)** | | | |
|---|---|---|---|---|---|---|---|---|
| Characteristics | Selected | SNPs dataset [a] | Microsatellites dataset [a] | DBLα types dataset | Selected | SNPs dataset | Microsatellites dataset | DBLα types dataset |
| Total population (N) | 215 | 157 | 192 | 172 | 200 | 200 | 200 | 193 |
| Age groups [b] | | | | | | | | |
| Children (1-10 years) | 81 (37.7) | 53 (33.8) | 73 (38.0) | 64 (37.2) | 86 (43.0) | 86 (43.0) | 86 (43.0) | 79 (40.9) |
| Adolescent (11-20 years) | 55 (25.6) | 45 (28.7) | 52 (27.1) | 48 (27.9) | 58 (29.0) | 58 (29.0) | 58 (29.0) | 58 (30.1) |
| Adults (>20 years) | 79 (36.7) | 59 (37.6) | 67 (34.9) | 60 (34.9) | 56 (28.0) | 56 (28.0) | 56 (28.0) | 56 (29.0) |
| Sex [b] | | | | | | | | |
| Female | 114 (53.0) | 87 (55.4) | 101 (52.6) | 90 (52.3) | 98 (49.0) | 98 (49.0) | 98 (49.0) | 94 (48.7) |
| Male | 101 (47.0) | 70 (44.6) | 91 (47.4) | 82 (47.7) | 102 (51.0) | 102 (51.0) | 102 (51.0) | 99 (51.3) |
| Microscopic *P. falciparum* median density [c] | 240 [120 – 1,460] (40 – 126,040) | 240 [120 – 1,560] (40 – 126,040) | 240 [120 – 2,020] (40 – 126,040) | 360 [120 – 2,880] (40 – 126,040) | 320 [120 - 1,190] (40 – 113,520) | 320 [120 - 1,190] (40 – 113,520) | 320 [120 - 1,190] (40 – 113,520) | 360 [160 – 1,280] (40 – 113,520) |
| *P. falciparum* median $MOI_{var}$ [d] | 2 [1 - 2] (1 – 4) | 2 [1 - 2] (1 – 4) | 1 [1 - 2] (1 – 4) | 2 [1 - 2] (1 – 4) | 1 [1 - 2] (1 – 3) | 1 [1 - 2] (1 – 3) | 1 [1 - 2] (1 – 3) | 1 [1 - 2] (1 – 3) |

N = number microscopically positive *P. falciparum* isolates; SNP = single nucleotide polymorphism

[a] Of the 215 isolates selected pre-IRS, a slightly different subset of 200 isolates had to be used for the SNP and microsatellite genotyping due to isolate availability. However, between these two datasets, 92.5% (N = 185) isolates genotyped were the same.

[b] Data reflect the number (% (n/N)) of participants sampled that were microscopically positive for *P. falciparum* (including mixed *P. falciparum* infections).

[c] Median density for all microscopically positive *P. falciparum* isolates (including mixed *P. falciparum* infections) (value/µl, interquartile range [IQR], minimum and maximum density (min-max)).

[d] Median $MOI_{var}$ for all microscopically positive *P. falciparum* isolates (value, interquartile range [IQR], minimum and maximum MOI(min-max)).

**Table S3.** SNP genotyping success for the pre-IRS and post-IRS. Note that the four SNP loci highlighted in grey had a call rate (i.e., genotyping success) < 80% in either survey (i.e., A4, A10, B10, and B12) and were removed resulting in 20 SNP loci being used for the analyses.

| | | | Genotyping Success (%) | |
|---|---|---|---|---|
| **Locus** | **SNP ID#** | **Chr.** | **Pre-IRS (October 2012) (N = 161)** | **Post-IRS (October 2015) (N = 200)** |
| A1 | Pf_01_000130573 | 1 | 94.4 | 88.0 |
| B1 | Pf_01_000539044 | 1 | 98.1 | 97.5 |
| A2 | Pf_02_000842803 | 2 | 91.3 | 98.5 |
| B2 | Pf_04_000282592 | 4 | 98.8 | 99.0 |
| A3 | Pf_05_000931601 | 5 | 99.4 | 95.0 |
| B3 | Pf_06_000145472 | 6 | 98.8 | 96.0 |
| A4 | Pf_06_000937750 | 6 | 73.3 | 93.0 |
| B4 | Pf_07_000277104 | 7 | 99.4 | 99.5 |
| A5 | Pf_07_000490877 | 7 | 93.2 | 94.0 |
| B5 | Pf_07_000545046 | 7 | 99.4 | 97.5 |
| A6 | Pf_07_000657939 | 7 | 89.4 | 96.5 |
| B6 | Pf_07_000671839 | 7 | 90.7 | 98.5 |
| A7 | Pf_07_000683772 | 7 | 90.1 | 96.5 |
| B7 | Pf_07_000792356 | 7 | 100 | 98.5 |
| A8 | Pf_07_001415182 | 7 | 100 | 100 |
| B8 | Pf_08_000613716 | 8 | 91.9 | 100 |
| A9 | Pf_09_000634010 | 9 | 82.0 | 99.5 |
| B9 | Pf_10_000082376 | 10 | 96.3 | 87.5 |
| A10 | Pf_10_001403751 | 10 | 0.0 | 100 |
| B10 | Pf_11_000117114 | 11 | 64.6 | 100 |
| A11 | Pf_11_000406215 | 11 | 100 | 100 |
| B11 | Pf_13_000158614 | 13 | 100 | 100 |
| A12 | Pf_13_001429265 | 13 | 98.8 | 94.0 |
| B12 | Pf_14_000755729 | 14 | 66.5 | 100 |
| **Median** | | | **95.3** | **98.5** |

**Table S4.** The number of isolates with double-allele calls (DAC), major and minor alleles, and minor allele frequencies (MAF) for each of the 20 SNP loci genotyped using the "cleaned infections" pre-IRS dataset (N=157). Note the loci highlighted in grey had a MAF ≤ 10% (0.10).

| | | Pre-IRS (October 2012) (N = 157) | | | |
|---|---|---|---|---|---|
| **Locus** | **Chr.** | **N** | **DAC** | **Alleles [a]** | **MAF (excl. DAC) [b]** |
| A1 | 1 | 149 | 0 | C (T) | 0.25 |
| B1 | 1 | 154 | 0 | A (G) | 0.05 |
| A2 | 2 | 144 | 23 | C (T) | 0.25 |
| B2 | 4 | 155 | 18 | C (T) | 0.38 |
| A3 | 5 | 157 | 0 | C (G) | 0.01 |
| B3 | 6 | 156 | 0 | C (G) | 0.10 |
| B4 | 7 | 156 | 103 | A (G) | 0.38 |
| A5 | 7 | 147 | 22 | A (T) | 0.13 |
| B5 | 7 | 156 | 0 | T (C) | 0.01 |
| A6 | 7 | 143 | 56 | T (C) | 0.34 |
| B6 | 7 | 145 | 47 | G (A) | 0.47 |
| A7 | 7 | 145 | 32 | C (T) | 0.48 |
| B7 | 7 | 157 | 0 | A | 0.00 |
| A8 | 7 | 157 | 0 | C (A) | 0.01 |
| B8 | 8 | 148 | 9 | C (A) | 0.19 |
| A9 | 9 | 132 | 16 | C (A) | 0.46 |
| B9 | 10 | 151 | 0 | T (A) | 0.01 |
| A11 | 11 | 157 | 0 | C (A) | 0.09 |
| B11 | 13 | 157 | 6 | T (C) | 0.03 |
| A12 | 13 | 155 | 0 | G (T) | 0.03 |

N = Number of isolates with SNP data, ambiguous or missing allele calls (i.e., X) removed; DAC = Double-allele calls
[a] Alleles are presented as major (minor)
[b] Minor allele frequency (MAF) calculated excluding those isolates with DAC at the SNP locus

**Table S5.** The number of isolates with double-allele calls (DAC), major and minor alleles, and minor allele frequencies (MAF) for each of the 20 loci genotyped using the "cleaned infections" post-IRS (N=200) dataset. Note the loci highlighted in grey had a MAF ≤ 10% (0.10).

| | | | Post-IRS (October 2015) (N = 200) | | |
|---|---|---|---|---|---|
| **Locus** | **Chr.** | **N** | **DAC** | **Alleles [a]** | **MAF (excl. DAC) [b]** |
| A1 | 1 | 176 | 0 | C (T) | 0.45 |
| B1 | 1 | 195 | 0 | A (G) | 0.14 |
| A2 | 2 | 197 | 0 | C (T) | 0.05 |
| B2 | 4 | 198 | 32 | C (T) | 0.37 |
| A3 | 5 | 190 | 0 | C (G) | 0.03 |
| B3 | 6 | 192 | 4 | C (G) | 0.37 |
| B4 | 7 | 199 | 158 | G (A) | 0.46 |
| A5 | 7 | 188 | 6 | A (T) | 0.40 |
| B5 | 7 | 195 | 0 | T (C) | 0.03 |
| A6 | 7 | 193 | 17 | T (C) | 0.08 |
| B6 | 7 | 197 | 38 | G (A) | 0.15 |
| A7 | 7 | 193 | 0 | T (C) | 0.43 |
| B7 | 7 | 197 | 0 | A (C) | 0.04 |
| A8 | 7 | 200 | 0 | C (A) | 0.20 |
| B8 | 8 | 200 | 0 | C (A) | 0.06 |
| A9 | 9 | 199 | 0 | T (C) | 0.17 |
| B9 | 10 | 175 | 2 | T (A) | 0.06 |
| A11 | 11 | 200 | 0 | C (A) | 0.08 |
| B11 | 13 | 200 | 19 | T (C) | 0.07 |
| A12 | 13 | 188 | 0 | G (T) | 0.11 |

N = Number of isolates with SNP data, ambiguous or missing allele calls (i.e., X) removed; DAC = Double-allele calls
[a] Alleles are presented as major (minor)
[b] Minor allele frequency (MAF) calculated excluding those isolates with DAC at the SNP locus

**Table S6.** Genetic relatedness among the pre- and post-IRS surveys. The pairwise allele sharing ($P_{AS}$; SNPs and microsatellites) and pairwise type sharing (PTS; DBLα types) statistics were used to assess the extent of sharing (or relatedness) between isolates (Figure 4). While all of the isolates successfully *var*coded were used to calculate PTS, only the "monoclonal infections" with complete multilocus infection haplotypes (i.e., no missing genotype data) were used to calculate $P_{AS}$ (Figure 2).

| Genetic markers | Pre-IRS (October 2012) | | Post-IRS (October 2015) | | Pre-IRS vs. Post-IRS *p-value* [a] |
|---|---|---|---|---|---|
| | **N** | median $P_{AS}$ or PTS [IQR] | **N** | median $P_{AS}$ or PTS [IQR] | |
| SNP | 34 | 0.750 [0.700 - 0.800] | 68 | 0.750 [0.700 - 0.850] | < 0.001 |
| Microsatellites | 81 | 0.200 [0.100 - 0.300] | 84 | 0.200 [0.100 - 0.300] | < 0.001 |
| DBLα types | 172 | 0.020 [0.013 – 0.034] | 193 | 0.017 [0.000 -0.030] | < 0.001 |

N = Number of isolates; IQR = interquartile range
[a] Mann-Whitney U test (Wilcoxon rank sum test) was used to compare the $P_{AS}$ and PTS distributions between the pre- and post-IRS surveys.

**Table S7.** Genetic relatedness among the pre- and post-IRS surveys. Pairwise type sharing (PTS, DBLα types) scores were calculated among the isolate repertoires in each age group pre- and post-IRS (Figure 5) using all isolates with *var*coding data (Table S1).

| **Age group** | **Pre-IRS (October 2012)** median PTS [IQR] | **Post-IRS (October 2015)** median PTS [IQR] | **Pre-IRS vs. Post-IRS *p-value*** [a] |
|---|---|---|---|
| Children (1-10 years) | 0.039 [0.027 - 0.052] | 0.024 [0.014 - 0.037] | < 0.001 |
| Adolescent (11-20 years) | 0.032 [0.020 - 0.046] | 0.023 [0.013 - 0.035] | < 0.001 |
| Adults (>20 years) | 0.022 [0.013 - 0.035] | 0.019 [0.000 - 0.031] | < 0.001 |

IQR = interquartile range
[a] Mann-Whitney U test (Wilcoxon rank sum test) was used to compare the PTS distributions in each age group between the pre- and post-IRS surveys.

**References**

1. Tiedje KE, Oduro AR, Bangre O, Amenga-Etego L, Dadzie SK, Appawu MA, et al. Indoor residual spraying with a non-pyrethroid insecticide reduces the reservoir of Plasmodium falciparum in a high-transmission area in northern Ghana. PLOS Glob Public Heal. 2022;2(25):e0000285.
2. Rask TS, Hansen D, Theander TG, Pedersen AG, Lavstsen T, Gorm Pedersen A, et al. Plasmodium falciparum erythrocyte membrane protein 1 diversity in seven genomes - divide and conquer. PLoS Comput Biol. 2010;6(9):e1000933.
3. Daniels R, Volkman SK, Milner DA, Mahesh N, Neafsey DE, Park DJ, et al. A general SNP-based molecular barcode for Plasmodium falciparum identification and tracking. Malar J. 2008;7:223.
4. Watson OJ, Okell LC, Hellewell J, Slater HC, Unwin HJT, Omedo I, et al. Evaluating the Performance of Malaria Genetics for Inferring Changes in Transmission Intensity Using Transmission Modeling. Mol Biol Evol. 2021;38(1):274–89.
5. Daniels RF, Schaffner SF, Wenger E a., Proctor JL, Chang H-H, Wong W, et al. Modeling malaria genomics reveals transmission decline and rebound in Senegal. PNAS. 2015;112(22):7067–72.
